# Hemorrhagic Transformation After Ischemic Stroke: Mechanisms and Management

**DOI:** 10.3389/fneur.2021.703258

**Published:** 2021-11-30

**Authors:** Ji Man Hong, Da Sol Kim, Min Kim

**Affiliations:** ^1^Department of Neurology, Ajou University School of Medicine, Ajou University Medical Center, Suwon-si, South Korea; ^2^Department of Biomedical Science, Ajou University School of Medicine, Ajou University Medical Center, Suwon-si, South Korea

**Keywords:** cerebral hemorrhage, stroke, acute, hemorrhagic transformation (HT), risk factors, reperfusion

## Abstract

Symptomatic hemorrhagic transformation (HT) is one of the complications most likely to lead to death in patients with acute ischemic stroke. HT after acute ischemic stroke is diagnosed when certain areas of cerebral infarction appear as cerebral hemorrhage on radiological images. Its mechanisms are usually explained by disruption of the blood-brain barrier and reperfusion injury that causes leakage of peripheral blood cells. In ischemic infarction, HT may be a natural progression of acute ischemic stroke and can be facilitated or enhanced by reperfusion therapy. Therefore, to balance risks and benefits, HT occurrence in acute stroke settings is an important factor to be considered by physicians to determine whether recanalization therapy should be performed. This review aims to illustrate the pathophysiological mechanisms of HT, outline most HT-related factors after reperfusion therapy, and describe prevention strategies for the occurrence and enlargement of HT, such as blood pressure control. Finally, we propose a promising therapeutic approach based on biological research studies that would help clinicians treat such catastrophic complications.

## Introduction

Hemorrhagic transformation refers to hemorrhagic infarction that occurs after venous thrombosis or arterial thrombosis and embolism (1, 2). Autopsy studies have reported an HT rate of 18–42% in acute ischemic stroke due to arterial occlusion (1, 3). The frequency of HT has been reported mainly in clinical studies using brain imaging modalities, such as computed tomography (CT) or magnetic resonance imaging (MRI), rather than pathological studies (4). Therefore, prior to considering the frequency of occurrence of HT, we need to understand the imaging and clinical definitions of HT. Although rates of HT in ischemic stroke have been reported, more than half of all cerebral infarctions demonstrate certain stages of HT (5).

The radiographic definition of HT is generally classified by the European Cooperative Acute Stroke Study (ECASS) (6). On CT scans, the severity of HT is divided into two stages: hemorrhagic infarction (HI) and parenchymal hemorrhage (PH) with or without mass effect. Each stage is divided into two subtypes (7). Each characteristic is presented in Table 1 (8).

**Table 1:** Characteristics of hemorrhagic transformation (HT) according to European Cooperative Acute Stroke Study (ECASS) 2 (8).

**Types of HT**	**Mass effect**	**Definition**
Hemorrhagic infarction-1 (HI-1)	Absence of mass effect	Small petechial bleeding along the margins of the infarcted area
Hemorrhagic infarction-2 (HI-2)		Confluent petechial bleeding within the infarcted area
Parenchymal hemorrhage-1 (PH-1)	Mildmass effect	Hematoma in <30% of the infarcted area
Parenchymal hemorrhage-2 (PH-2)	Definite mass effect	Hematoma in more than 30% of the infarcted area

With recent advances in intravenous (9) or endovascular (10) reperfusion therapies for acute ischemic stroke (11), stroke physicians need to deepen their understanding of cerebral hemorrhagic complications. Although the overall risks of complications have been well-documented in various randomized controlled trials (RCTs) of reperfusion therapies (12), the mechanisms underlying cerebral hemorrhage or hematoma after stroke in individual patients remain poorly understood. Intracranial bleeding after acute ischemic stroke has a significant impact on patient outcomes (13, 14), and controlling the risk of bleeding plays an important role in determining whether to proceed with recanalization (15). Large parenchymal hematomas and symptomatic intracerebral hemorrhage (sICH) are the most feared, tend to have a high mortality rate, and appear in up to 6% of patients after intravenous thrombolysis (16). In addition, infarction evolution with HT can lead to significant neurological deterioration (17–19). The frequency of HT is associated with different factors, such as epidemiological factors (e.g., age, pre-stroke treatment, and conditions), characteristics of the infarct (size of ischemic core and timing of follow-up), reperfusion techniques in the acute phase (intravenous thrombolysis, mechanical thrombectomy, or combined), radiological diagnosis (CT or MRI techniques), and use of antithrombotics after the acute phase (20–22).

## Clinical Presentations, Histopathology, and Radiologic Features

Various criteria have been applied to define whether a hemorrhagic infarction is symptomatic; however, only parenchymal hematomas have been reported to be consistently linked to worsening and long-term deterioration (23). Many cases of HT, including most petechial hemorrhages, are asymptomatic (24). Only sICH (parenchymal hematoma) appears to be clinically evident and often exhibits rapid neurological deterioration (25). In untreated patients, HT rarely occurs during the first 6 h. It usually appears in the first few days, most within 4 days of infarction (26, 27). Patients who have undergone acute treatment with thrombolysis or thrombectomy usually experience bleeding 24 h after stroke onset (early HT) (28).

Pathologists have traditionally called petechial HT “red softening.” Petechial HT is considered to be due to (a) insufficient perfusion from adjacent collateral vessels or (b) reperfusion of infarcted tissues with weakened vessels (extravasation) (29). The former explains why HT occurs in patients with permanently occluded vessels (30), while the latter explains why the proportion of patients with HT is higher in those who receive reperfusion therapy than in those who do not receive reperfusion therapy (31).

**Figure 1:**
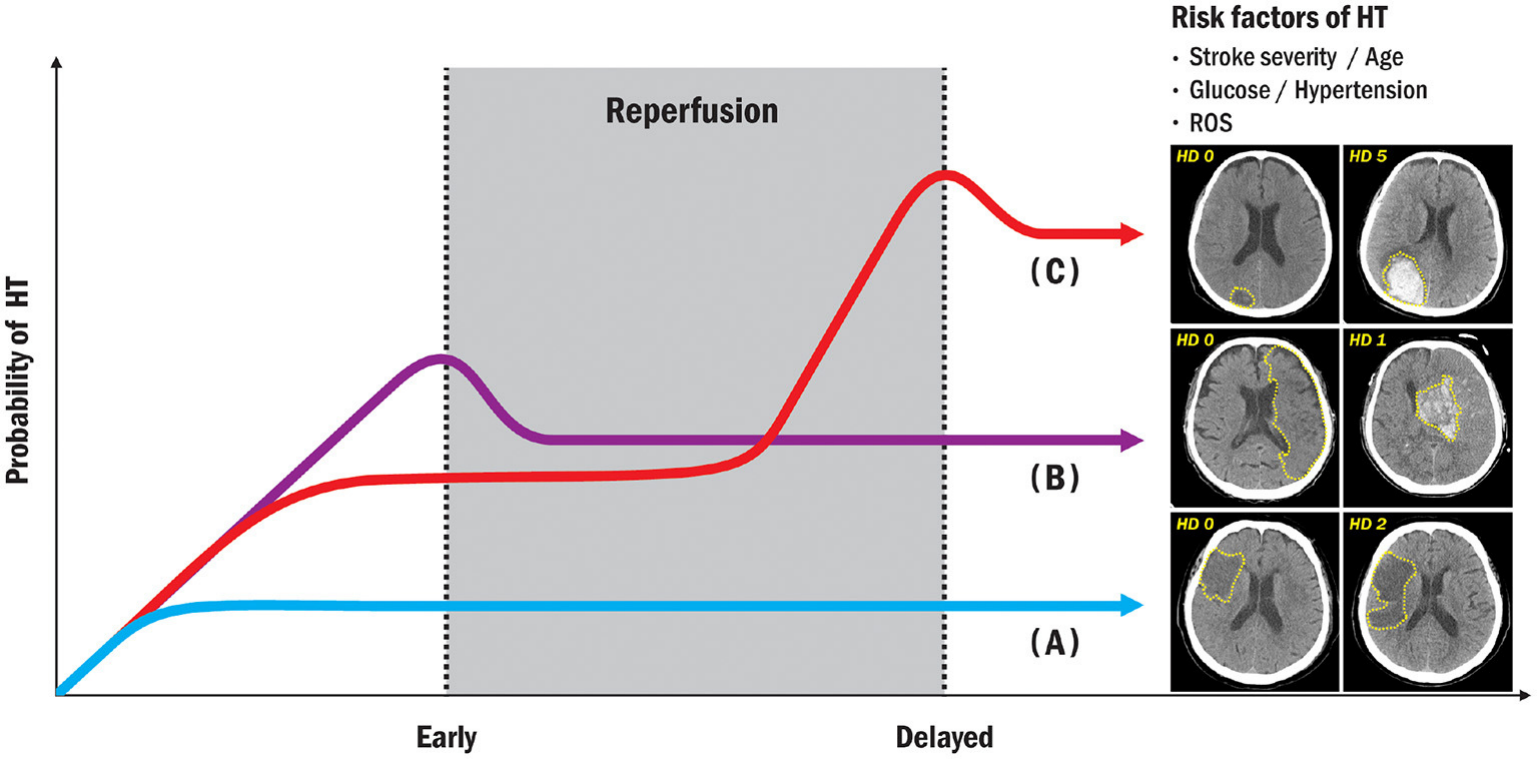
Illustration showing correlation between HT and reperfusion time after ischemic stroke. Under the risk factors commonly associated with HT; **(A)** no HT (no bleeding regardless of reperfusion); **(B)** early HT (definite bleeding usually 6–24 h after stroke); **(C)** delayed HT (definite bleeding usually more than 24 h after ischemic stroke). HT, hemorrhagic transformation; ROS, reactive oxygen species.

Figure 1 shows the relationship between HT probability and reperfusion (R) after ischemic stroke. The radiologic features differ from those of petechial hemorrhagic infarction and parenchymal hemorrhage (19). Petechial hemorrhagic infarction usually appears as tiny punctate regions in the hemorrhage and is often not individually resolved (32). In parenchymal hematomas or hemorrhage, radiological features on both CT and MRI, which combine the features of ischemic infarction and cerebral hemorrhage, overlap (33).

## Mechanisms of HT After Ischemic Stroke

**Figure 2:**
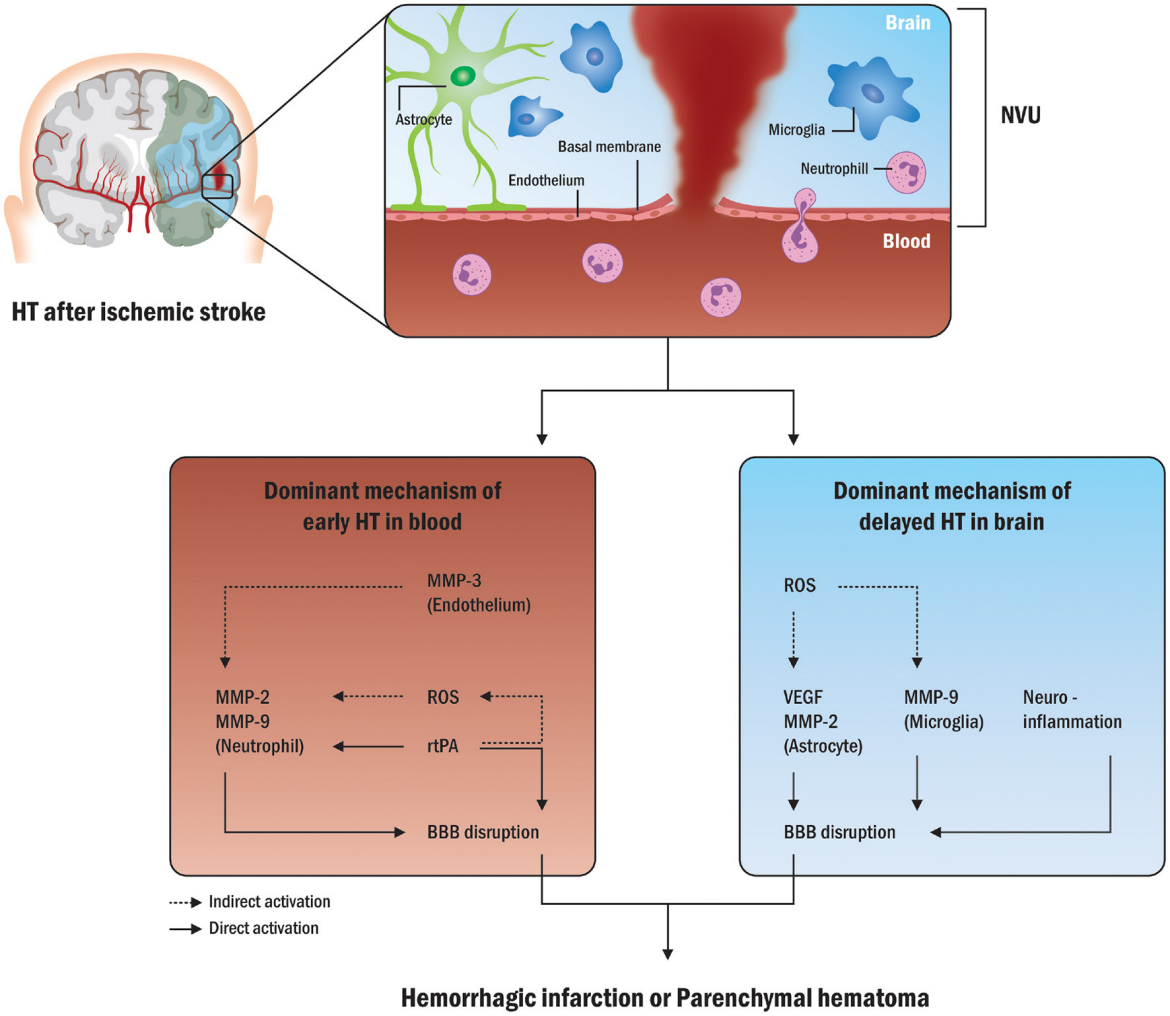
Possible mechanisms in early and delayed HT. The disruption of the BBB is a common pathway in HT formation following acute ischemic stroke. Various molecules from neutrophils and peripheral blood in possible processes in early HT are mainly associated with HT after ischemic stroke. Exogenous tPA can also increase MMP-9 levels by activating neutrophils and increasing MMP-2 levels. Conversely, in possible processes for delayed HT, the brain tissue is a major source of MMP-9 within the first 18–24 h following stroke, and endogenous tPA can act on endothelial cells to increase MMP-2 release from astrocytes as well as MMP-9 release from microglia. HT, hemorrhagic transformation; NVU, neurovascular unit; MMP, matrix-metalloproteinase; ROS, reactive oxygen species; tPA, tissue plasminogen activator; BBB, blood-brain barrier; VEGF, vascular endothelial growth factor.

The blood–brain barrier (BBB) is a physiological barrier between the brain parenchyma and brain circulation that nourishes brain tissue, filters various substances from the brain to the blood, and protects the brain (34, 35). The BBB is composed of endothelial cells, basement membrane, pericytes, and astrocytes, collectively referred to as the neurovascular unit and linked to circulating peripheral blood cells (36, 37). Early disruption of the BBB plays a pivotal role in HT formation during acute ischemic stroke (38). Leukocyte types and various molecules are associated with HT after ischemic stroke (39). Neutrophils and brain tissue are major sources of matrix-metalloproteinase-9 (MMP-9) within the first 18–24 h after stroke (28, 40). Intravenous infusion of exogenous tissue plasminogen activator (tPA) can increase MMP-9 levels by activating neutrophils (41), and endogenous tPA can increase MMP-3 levels by acting on endothelial cell lipoprotein receptor protein (LRP) (42), and can increase MMP-2 levels by activating platelet-derived growth factor-CC as a trigger *via* astrocyte platelet-derived growth factor receptor A (43). Figure 2 shows a possible mechanism for early vs. delayed HT.

Theoretically, cerebral infarction does not occur until the cerebral blood flow reaches a minimum threshold, where oxygen and glucose cannot be sufficiently guaranteed (44). As in other organs, infarcted cerebral tissue tends to bleed, and cerebral hemorrhage can lead to severe neurological deterioration (22). Mechanisms related to HT can be considered from various perspectives, such as histological changes, vascular occlusion, collateral circulation, BBB disruption, and infarct size (45, 46).

Acute cerebral ischemia leads to considerable damage to capillary cells, which causes an increase in vascular permeability and extravasation of blood in the brain parenchyma (47, 48). The two main factors described in this process are oxidative stress and reperfusion injury, which cause damage to blood vessels through various injury mechanisms, such as inflammation, leukocyte infiltration, vascular activation, and extracellular proteolysis (49, 50). The consequences are destruction of the basal lamina and endothelial tight junctions (51). Among the molecular processes involved, MMP-9 has been shown to play an important role in the destruction of basal lamina type IV collagen (52, 53). Destruction of the basal lamina leads to leakage of macromolecules into interstitial fluids in the central nervous system (54). In contrast to cytotoxic edema (cell death from ionic pump failure), the resulting ionic gradient causes interstitial edema, known as “vasogenic edema” (55). Vasogenic edema can lead to lesions in adjacent tissues. Therefore, this mechanism can worsen, causing malignant infarction, resulting in fatal consequences and high risk of HT (56).

Reperfusion can trigger harmful cascades, such as oxidative stress, suppression of protein synthesis, platelet activation, activation of the complement system, leukocyte infiltration, basal lamina disruption, and eventual cerebral cell death in the central nervous system (57–60). Reperfusion injury alone seems to be enough to cause fatal hematoma, but all ischemic strokes with tissue reperfusion do not cause hematoma (29). Fragments of thrombus with a large thrombotic burden can contribute to bleeding complications in the delayed phase (> 24 h) after acute stroke. Fragmentation of a large thrombus can lead to distal migration and damage to the vascular bed (61).

In summary, the development of HT after stroke involves multiple interconnected pathological processes from peripheral blood cells to neurovascular units, such as hyperactive ischemic cascades with increased MMP levels, excessive levels of ROS, coagulopathy, BBB breakdown, and reperfusion injury.

## Factors Associated With HT After Ischemic Stroke

Although the usefulness of these HT-related factors may be limited in clinical practice, some factors predict HT. Given the fibrinolytic or antithrombotic therapy in acute ischemic stroke settings, imaging techniques, and predictive biomarkers can help screen specific patients at increased risk of HT as a group of particular interest (62–64). Advances in the use of neuroimaging and composite scores can lead to more personalized approaches for HT prediction, but various factors should be considered when drawing conclusions that may affect the timing of HT detection (16, 28). This point can be influenced by the accuracy of imaging modalities used, such as CT or MRI with or without gradient-echo or susceptibility-weighted sequences (65), which are more sensitive to the detection of blood products (64). Factors associated with HT after ischemic stroke are shown in detail in Table 2.

**Table 2:** Various associated factors with HT.

**Associated factors**	**High risk**	**Low risk**
**Clinical features**		
Age (21)	Old	Young
Sex (21)	Male	Female
Weight (66)	Obese	Normal weight
Temperature (67)	Fever	Normothermia
Glucose (68)	Hyperglycemia	Normoglycemia
Blood pressure (68)	Hypertensive	Normotensive
Variability of blood pressure (69)	Yes	No
Stroke severity (21)	Severe stroke (≥22 on NIHSS)	Mild stroke (1–5 on NIHSS)
Size/type of infarct (21)	Large/embolic territorial (MCA, ACA, PCA, cerebellar)	Small/lacunar or small vesseldisease
Atrial fibrillation (21)	Yes	No
Congestive heart failure (22)	Yes	No
Renal impairment (70)	Yes	No
Previous stroke (21)	Yes	No
Diabetes (21)	Yes	No
Platelet count (16)	Low	No
Previous antiplatelet treatment (68)	Yes	No
OTT (66)	Late (≥ 180 min)	Early (< 180 min)
ERT (71)	Late (> 6 h)	Early (≤ 6 h)
**Biochemical factors**		
MMP-9/c-Fn (70)	High	Low
Fibrinogen (16)	Low	High
Ferritin (28)	High	Low
S100B (72)	High	Low
TAFI (73)	High	Low
PAI-1 (73)	Low	High
VAP-1/SSAO activity (70)	High	Low
APC (28)	High	Low
PDGF-CC (74)	High	Low
**Genetics**		
Leukocyte mRNA (MCFD2, VEGI/AREG, MARCH7, SMAD4) (75)	Low/High	High/Low
A2M (76)	High	Low
Factor FXII (76)	Low	High
Factor FXIII V34L (77)	High	Low
**Imaging findings**		
Early signs of ischemia (21)	Yes	No
Focal hypodensity, edema, mass effect on baseline (20)	Yes	No
Leukoaraiosis (22)	Yes	No
BBB permeability (16)	Yes	No
Areas of hypoperfusion on CTP (78)	Yes	No
HARM (79)	Yes	No
MRI enhancement pattern (63)	Yes	No
Collateral flow (29)	Low	High
ADC value (80)	Low	High
Cerebral blood flow or volume (28)	High	Low
Infarct volume on DWI (25)	Large	Small
**Composite rating scores**		
HAT (0-5 points) (81)	High	Low
MSS (0-4 points) (82)	High	Low
SITS-SICH (0-12 points) (66)	High	Low
SEDAN (0-5 points) (83)	High	Low
GRASPS GWTG (0–101 points) (70)	High	Low
SPAN-100 (0–1 points) (84)	High	Low
THRIVE (0–9 points) (85)	High	Low

*NIHSS, National Institutes of Health Stroke Scale; MCA, middle cerebral artery; ACA, anterior cerebral artery; PCA, posterior cerebral artery; OTT, onset to treatment; ERT, endovascular recanalization therapy; MMP-9, matrix metalloproteinase-9; c-Fn, cellular fibronectin; S100B, S100 calcium-binding protein B; TAFI, thrombin activatable fibrinolysis inhibitor; PAI-1, plasminogen activator inhibitor; VAP-1, vascular adhesion protein-1; SSAO, semicarbazide-sensitive amine oxidase; APC, activated protein c; PDGF-CC, platelet-derived growth factor-cc; mRNA, messenger ribonucleic acid; MCFD2, multiple coagulation factor deficiency protein 2; VEGI, vascular endothelial growth factor; AREG, Amphiregulin; MARCH7, membrane-associated RING-CH-type finger 7; SMAD4, smad family member 4; A2M, alpha-2-macroglobulin; BBB, blood-brain-barrier; CTP, computed tomography perfusion; HARM, hyperintense acute injury marker; MRI, magnetic resonance imaging; ADC, apparent diffusion coefficient; DWI, diffusion-weighted imaging; HAT, hemorrhage after thrombolysis; MSS, multicenter stroke survey; SITS-SICH, safe implementation of treatments in stroke symptomatic intracerebral hemorrhage; SEDAN, blood sugar, early infarct signs, hyperdense cerebral artery sign, age, NIHSS; GRASPS GWTG, glucose at presentation, race, age, sex, systolic blood pressure at presentation, and severity of stroke at presentation (NIHSS)-Get with the Guidelines; SPAN-100, stroke prognostication using age and NIHSS; THRIVE, totaled health risks in vascular events*.

## Reversal of Coagulopathy With Various Agents

Although coagulopathy correction remains the mainstay of treatment after tPA infusion, no specific agent has been found to be most effective in dealing with fatal HT expansion, which includes sICH (86). In patients with sICH, which occurs within 36 h after tPA infusion, there are several suggestions that can be considered depending on the mechanisms of action of reversal agents (87). The details are listed in Table 3.

**Table 3:** Potential reversal agents for treatment of HT.

**Reversal agent**	**Suggested dose**	**A promising treatment group**	**Adverse effects**
Cryo-precipitate	10 U	All sICH patients	Lack of pathogen inactivation, risk of transfusion related lung injury, and delay in obtaining the solution
Platelets	6–8 U	MostsICH patients (except for patients with thrombocytopenia, which platelet count <100,000/μL)	Lack of pathogen inactivation, risk of transfusion-related lung injury
PCC	20–40 mL	sICH patients on warfarin treatment before alteplase administration (adjunct treatment to cryo-precipitate)	Risk of thrombotic complication
FFP	12 mL/kg	sICH patients on warfarin treatment before alteplase administration but cannot treat PCC (adjunct treatment to cryoprecipitate)	Risk of thrombotic complications, and volume overload
Vitamin K	5–10 mg	sICH patients on warfarin treatment before alteplase administration	Risk of anaphylaxis
Antifibrinolytic agent	Amicar: 1–4 g/h TXA: 10 mg/kg	All sICH patients (especially, those who decline blood products)	Risk of thrombotic complications
rFVIIa	20–160 μg/kg	Unclear	Risk of thrombotic complication

*sICH, symptomatic intracerebral hemorrhage; PCC, prothrombin complex concentrates; FFP, fresh frozen plasma; Amicar, aminocaproic acid; TXA, tranexamic acid; rFVIIa, recombinant factor VIIa*.

## Prevention of HT Expansion

Hematoma expansion or sICH is a major predictor of death and disability in patients with acute stroke with HT (88). Therefore, in addition to aggressive reversal of coagulopathy, other strategies to prevent hematoma expansion may be needed as therapeutic targets in sICH.

Elevation and variability in blood pressure have been linked to the risk of hematoma enlargement in patients with spontaneous ICH in observational studies (89). In patients with spontaneous intracerebral hemorrhage, studies have shown that intensive control of systolic blood pressure is relatively safe to lower to 140 mmHg, but this that measure had no apparent effect compared with the systolic blood pressure target of 180 mmHg (90). Although the optimal target for blood pressure control in sICH is still unclear, the treatment goal is to supply adequate blood flow to the ischemic area, reduce the pressure on the brain with autoregulation impairment, and eventually reduce the risk of hematoma expansion (91). However, the effects of BP on hematoma enlargement are for primary intracranial hemorrhage and are independent of those in patients with HT after ischemic stroke. Nonetheless, the recent Enhanced Control of Hypertension and Thrombolysis Stroke Study (ENCHANTED) trial has shown that intensive blood pressure control potentially reduces the risk of major intracranial hemorrhage in patients with acute ischemic stroke receiving intravenous thrombolytic therapy (92).

Patients with acute ischemic stroke may be at risk of additional ischemia, especially in a low blood pressure environment, if occluded vessels are not reopened following thrombolytic therapy or mechanical thrombectomy. Although several studies have linked poor neurological outcomes to decreased mean arterial pressure (93), Rasmussen et al. only included patients whose blood pressure was measured during endovascular procedures or intravenous alteplase infusion procedures, and the results were stratified according to the presence of sICH (94).

One study related to thrombolysis and blood pressure showed that decreased systolic blood pressure was associated with improved neurological outcomes and lower rates of sICH (86, 95). In the European Cooperative Acute Stroke Study II (ECASS II) clinical trial, higher systolic blood pressure was associated with worse functional outcomes and sICH (93). However, there was no clear evidence that lower blood pressure led to worse functional outcomes (87). In the presence of lethal HT after tPA infusion (especially parenchymal hemorrhage type 2), few data on blood pressure treatment are available, especially when compared with other types of HT. In tPA-related HT, healthcare providers should determine the target blood pressure and consider the severity of sICH, risk of bleeding enlargement, and risk of impending ischemia (96). Theoretically, with incomplete recanalization, higher blood pressure targets may be needed to maintain adequate collateral blood flow to the ischemic bed and reduce the risk of infarct growth among patients with HI-1 and HI-2 (97). Under complete recanalization, strict blood pressure control measures may be reasonable (98).

Stricter blood pressure control may be more beneficial and less harmful for patients with parenchymal hematoma at higher risk of hematoma enlargement. Hematomas with smaller volumes are generally left untreated (99), and deep-seated (thalamus or brainstem) hemorrhages are usually not evacuated.

In summary, healthcare providers should determine blood pressure targets by weighing the risk of worsening ischemia based on the severity of hemorrhage and its risk of expansion. Patients with incomplete recanalization may need higher blood pressure targets to maintain sufficient blood flow to the ischemic bed and reduce the risk of infarct growth. Conversely, patients with complete recanalization may need strict blood pressure control to avoid impending HT.

## Blood Pressure Management After Thrombectomy for Preventing HT

Observational studies have shown an increased risk of HT in patients with high blood pressure and high variability in blood pressure, suggesting a close relationship between hemodynamics and HT (69). High variability in blood pressure has been considered a strong risk factor for cerebral edema and post-stroke HT, as rapid changes in blood pressure can easily rupture already damaged blood vessels due to ischemic insult (100, 101). Current guidelines recommend maintaining blood pressure below a fixed threshold of 180/105 mmHg for at least 24 h, regardless of thrombolytic or endovascular intervention (96, 102). A recent US study reported that a peak systolic BP of 158 mmHg in the first 24 h after endovascular therapy best dichotomized good and bad outcomes (103). A prospective randomized trial reported neutral results when determining whether a target systolic blood pressure (SBP) <130 mmHg after endovascular reperfusion can reduce the risk of intracranial hemorrhage (104). Therefore, lowering the post-reperfusion BP target can be considered to prevent reperfusion injury and promote tissue restoration in ischemic penumbra (98).

Cerebral autoregulation is the intrinsic dilative-constrictive capacity of the cerebral vasculature that preserves stable blood flow in the face of systemic blood pressure changes (105, 106). Autoregulatory capacity in acute stroke is crucial for maintaining stable blood flow to the ischemic penumbra and avoiding excessive hyperperfusion (107).

**Figure 3:**
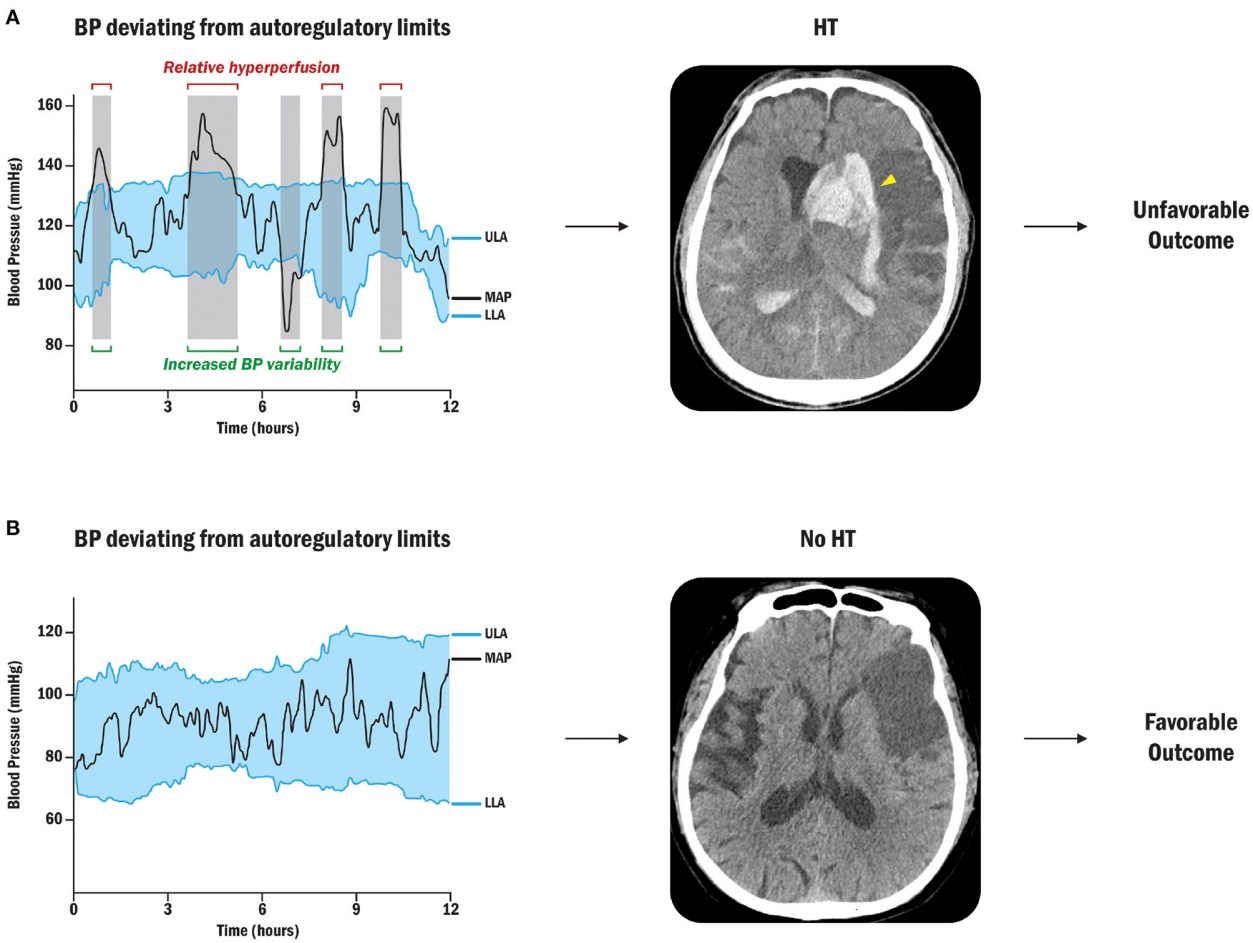
Sequential BP changes, cerebral autoregulation, and HT (91). **(A)** Patient with BP deviation from autoregulatory limits: Relative hyperperfusion above the upper limit of autoregulation may lead to HT and unfavorable outcomes. The yellow arrowhead points out the radiological HT after cerebral ischemic stroke. **(B)** Patient with acceptable BP fluctuations: Strictly controlled blood pressure within personalized limits of autoregulation can prevent secondary brain injury by protecting against HT after stroke. BP, blood pressure; ULA, upper limit of autoregulation; MAP, mean arterial pressure; LLA, lower limit of autoregulation.

Petersen et al. reported more longitudinal autoregulation modes, indicating dynamic autoregulatory failures up to 1 week after emergent large vessel occlusion (ELVO) strokes (108). Figure 3 shows the presence of HT within or above the autoregulatory limits due to fluctuations in blood pressure. This investigation showed that the autoregulatory parameter in the ipsilateral cerebral hemisphere was lower than that in the opposite hemisphere, indicating a decrease in the ability to buffer blood pressure fluctuations (91). In patients with stroke with cerebral autoregulation impairment, restoration tends to be delayed for up to 3 months, emphasizing the clinical relevance of autoregulation in stroke research (109, 110).

In summary, continuous optimization of blood pressure would be a good method for patients tailored to their own physiology, where hemodynamic management represents an appropriate and neuroprotective avenue for critically ill patients. Exceeding the upper limits of autoregulation may predispose patients to reperfusion injury, and maintaining blood pressure within autoregulatory limits may avoid bleeding complications while achieving favorable outcomes. Furthermore, trajectory analysis has the potential to provide more individualized hemodynamic management during and after thrombectomy procedures in intensive care settings.

## Medical Treatment and Neurosurgical Considerations

**Figure 4:**
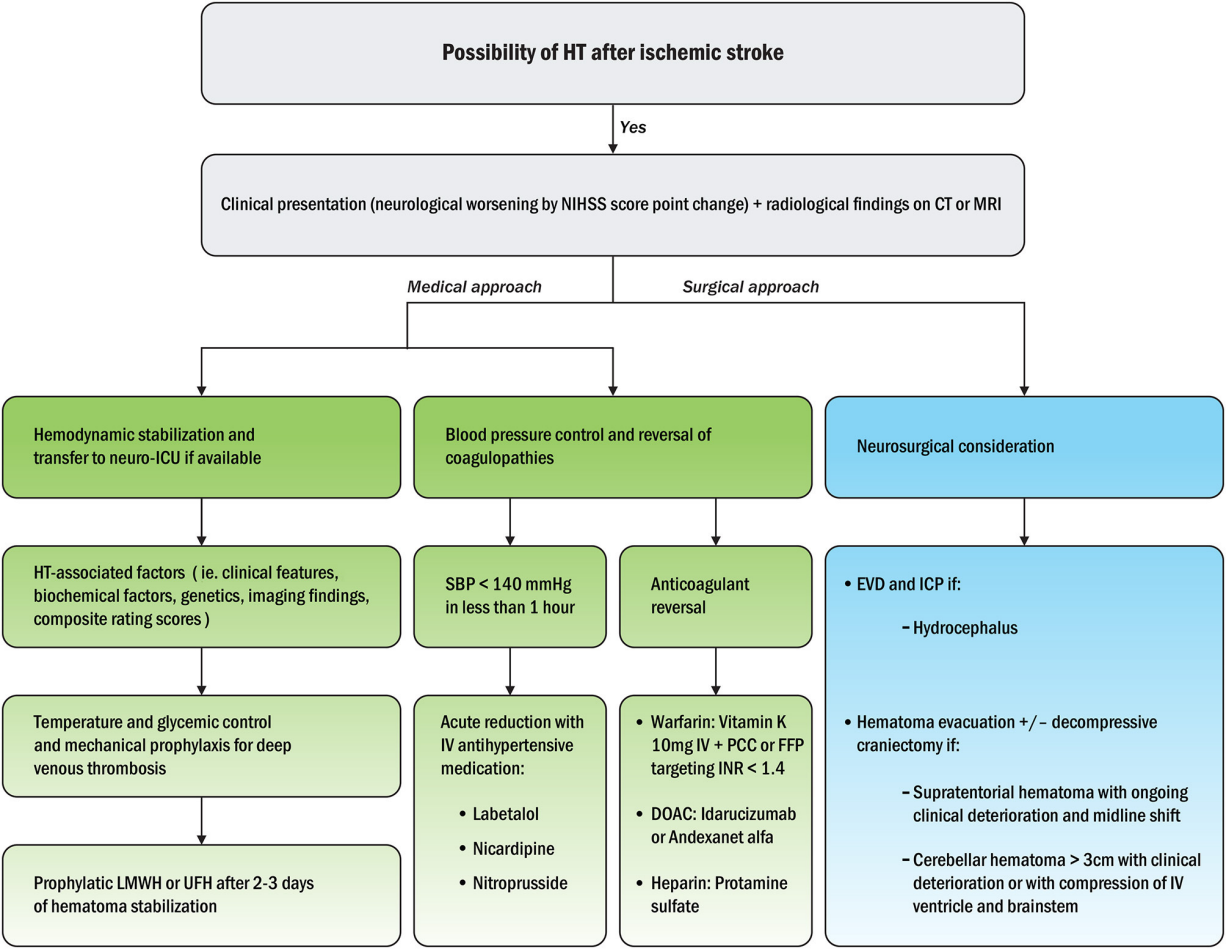
Treatment algorithm for appropriate medical and surgical approaches to HT after ischemic stroke (115). NIHSS, National Institutes of Health Stroke Scale; CT, computed tomography; MRI, magnetic resonance imaging; ICU, intensive care unit; LMWH, low molecular weight heparin; UFH, unfractionated heparin; SBP, systolic blood pressure; IV, intravenous; PCC, prothrombin complex concentrates; FFP, fresh frozen plasma; INR, international normalized ratio; DOAC, direct oral anticoagulants; EVD, external ventricular drainage; ICP, intracranial pressure.

Hemorrhagic transformation after ischemic stroke can be suspected based on clinical presentation (neurological worsening in National Institutes of Health Stroke Scale, NIHSS, score) and radiological findings within 48 h on CT or MRI (111, 112). First, hemodynamic stabilization should be performed, followed by transfer to a neuro-intensive care unit if available (113). To evaluate the mechanisms of HT, HT-associated factors, such as clinical features, biochemical factors, genetics, imaging findings, and composite rating scores, should be analyzed. Temperature and glycemic controls should then be performed, such as mechanical prophylaxis for deep vein thrombosis (86). Second, blood pressure control and correction of coagulopathies should be mainstays of HT treatment (114). Finally, neurosurgical considerations are needed as soon as possible if hazardous HT is suspected (115). A plausible algorithm for appropriate clinical approaches to HT after ischemic stroke is depicted in Figure 4.

It is challenging to construct evidence-based treatment algorithms for surgical interventions because of the lack of expected data to guide the therapeutic timing and surgical techniques to be implemented (116). Nonetheless, neurosurgical treatment can be considered in patients with sICH where outcomes can be improved despite ischemic injury. Indeed, the risks and benefits of rapid surgical decompression vs. iatrogenic injury must be carefully weighed in the setting of possible tPA-associated coagulopathy.

Neurosurgical treatment can also be considered in patients with supratentorial ICH who exhibit neurological deterioration, coma, significant midline shift, or elevated intracranial pressure refractory to medical treatment. The goal is to decompress the brain and reduce the impact of mass effect, malignant edema, and toxic blood byproducts (117). Open craniotomy can eliminate the compressive effect of a hematoma higher than 30 cm^3^ in volume from lobar, cerebellar, or surgically accessible basal ganglia hematomas (118, 119). However, this requires an incision through the cortex and white matter tracts along the path to the lesion. The clinical effectiveness of these interventions remains controversial (120, 121). Minimally invasive craniotomy and stereotactic hematoma evacuation are currently under investigation for spontaneous ICH and post-thrombolytic hemorrhage.

## Potential Therapeutic Interventions Along With Biological Research on HT

Since HT in acute ischemic stroke is radiologically diagnosed, the timing of HT detection is affected by the accuracy of imaging modalities, such as CT or MRI, with or without gradient-echo or susceptibility-weighted imaging sequences that are more sensitive to blood products (122).

Strong evidence exists that matrix metalloproteinases, especially MMP-9, play a pivotal role in the pathogenesis of the abnormal permeability of the BBB, an important culprit of HT (42, 52, 53). The use of drugs to block the release of MMP-9 is challenging. The phosphodiesterase-III inhibitor cilostazol prevented the development of HT, reduced brain edema, prevented endothelial injury *via* reduction of MMP-9 activity, and prevented the BBB from opening in an experimental model (123–125). Another drug, the broad-spectrum MMP inhibitor BB-94, reduced the risk and severity of HT in rats with homologous clot-induced middle cerebral artery occlusion compared with rats treated with intravenous tPA alone (41). The MMP-9 inhibitor minocycline reduced the risk of HT after tPA in animal models (126, 127), and the Minocycline to Improve Neurologic Outcome in Stroke (MINOS) human trials showed a decrease in plasma MMP-9 levels in patients treated with intravenous tPA (128, 129). Targeted temperature management may be an important step to mitigate HT after recanalization in patients with clinically malignant ELVO, as it reduces the metabolic rate and excessive free radical levels, protects the BBB by reducing MMP-2 or MMP-9 expression, and inhibits immune system responses (130, 131). Nevertheless, effective management of lethal HT requires further experimental studies and trials based on core molecular mechanisms.

## Conclusion

Symptomatic intracerebral hemorrhage is a life-threatening complication requiring emergent medical and surgical treatment in patients with acute ischemic stroke. Therefore, we need to understand possible mechanisms and treat this potentially serious complication with systematic algorithms in future stroke therapy.

## Author Contributions

JH performed roles of the conception of this review and wrote the manuscript, interpretation of data, and revising it critically for important intellectual content. DK performed roles of interpretation of data and drafting the article. MK performed roles of the acquisition of data. All authors contributed to the article and approved the submitted version.

## Conflict of Interest

The authors declare that the research was conducted in the absence of any commercial or financial relationships that could be construed as a potential conflict of interest.

## Publisher's Note

All claims expressed in this article are solely those of the authors and do not necessarily represent those of their affiliated organizations, or those of the publisher, the editors and the reviewers. Any product that may be evaluated in this article, or claim that may be made by its manufacturer, is not guaranteed or endorsed by the publisher.
